# Analysis of Molecular Imaging Biomarkers Derived from [^18^F]FDG PET/CT in mCRPC: Whole-Body Total Lesion Glycolysis (TLG) Predicts Overall Survival in Patients Undergoing [^225^Ac]Ac-PSMA-617-Augmented [^177^Lu]Lu-PSMA-617 Radioligand Therapy

**DOI:** 10.3390/cancers16203532

**Published:** 2024-10-19

**Authors:** Caroline Burgard, Fadi Khreish, Lukas Dahlmanns, Arne Blickle, Moritz B. Bastian, Tilman Speicher, Stephan Maus, Andrea Schaefer-Schuler, Mark Bartholomä, Sven Petto, Samer Ezziddin, Florian Rosar

**Affiliations:** 1 Department of Nuclear Medicine, Saarland University Medical Center, 66421 Homburg, Germany; fadi.khreish@klinikum-fulda.de (F.K.); s8lsdahl@stud.uni-saarland.de (L.D.); arne.blickle@uni-saarland.de (A.B.); moritz.bastian@uks.eu (M.B.B.); tilman.speicher@uks.eu (T.S.); stephan.maus@uks.eu (S.M.); andrea.schaefer@uks.eu (A.S.-S.); mark.bartholomae@uks.eu (M.B.); sven.petto@uks.eu (S.P.); samer.ezziddin@uks.eu (S.E.); florian.rosar@uks.eu (F.R.); 2 Department of Nuclear Medicine, Campus Fulda, University of Marburg, 36043 Fulda, Germany

**Keywords:** FDG, total lesion glycolysis, TLG, biomarker, mCRPC, PSMA, radioligand therapy, ^225^Ac, tandem RLT

## Abstract

**Background/Objectives:** The augmentation of [^177^Lu]Lu-PSMA-617 radioligand therapy by alpha emitting [^225^Ac]Ac-PSMA-617, known as the tandem therapy concept, is a promising escalating treatment option in advanced mCRPC. In this study, we evaluated the value of [^18^F]FDG PET/CT-derived molecular imaging biomarkers for predicting response and outcome to PSMA tandem RLT in *n* = 33 patients with insufficient response on [^177^Lu]Lu-PSMA-617 monotherapy. **Methods:** Six different molecular imaging parameters at baseline, i.e., before initiation of PSMA tandem RLT with respect to SUV_max_, SUV_peak_, SUV_5_, SUV_mean_, metabolic tumor volume (MTV), and total lesion glycolysis (TLG) were tested for association with response and overall survival (OS). **Results:** After the initiation of augmentation, 24.2% of patients with a previously insufficient response experienced partial remission, and 39.4% experienced stable disease. The median OS was 7 months (95% CI: 4–11 months). None of the tested parameters were able to predict the response (all *p* > 0.529). In contrast, the [^18^F]FDG PET/CT-derived whole-body molecular imaging parameter TLG was significantly (*p* = 0.029) associated with OS of patients undergoing [^225^Ac]Ac-PSMA-617 augmented [^177^Lu]Lu-PSMA-617 RLT after insufficient response to [^177^Lu]Lu-PSMA-617 monotherapy. **Conclusion:** Implementing [^18^F]FDG PET/CT in the management of PSMA-RLT in clinical practice may contribute to outcome prediction and provide a route to more individualized management in mCRPC.

## Simple Summary

Augmentation of prostate-specific membrane antigen (PSMA)-targeted radioligand therapy (RLT) by alpha emitting ^225^Ac, known as the tandem therapy concept, is a promising escalating treatment option in patients with advanced metastatic castration-resistant prostate cancer (mCRPC). The aim of this study was to analyze the value of imaging parameters in baseline [^18^F]FDG PET/CT for predicting response and outcome to PSMA tandem RLT in patients with insufficient response to the initial [^177^Lu]Lu-PSMA-617 monotherapy. The quantitative whole-body imaging biomarker total lesion glycolysis (TLG) was identified as a prognostic biomarker for overall survival (OS), while response could not be predicted by any of the tested parameters. Using [^18^F]FDG PET/CT in clinical practice could help predict outcomes and may provide more personalized care for mCRPC patients.

## 1. Introduction

Currently, prostate cancer (PC) is listed among the most abundant forms of tumor disease worldwide [1]. While in early-stage PC, the survival probability is still comparably high, the prognosis worsens with progression into the condition of metastatic castration-resistant prostate cancer (mCRPC), which presents resistance to castration by, e.g., androgen deprivation therapy or prostatectomy (ADT) [2,3,4,5]. Nonetheless, there are different therapy options for patients diagnosed with mCRPC, including treatment with novel androgen axis drugs (NAAD) [6,7], taxane-based chemotherapy [8,9], bone-seeking ^223^Ra [10], and PARP inhibitors [11]. However, a considerable share of patients show a progressive disease despite intensive treatment efforts.

In this scenario, radioligand therapy (RLT), targeting the prostate-specific membrane antigen (PSMA) is a favorable option for treatment. PSMA-RLT, using the beta emitter ^177^Lu in the form of [^177^Lu]Lu-PSMA-617, was recently approved by the Food and Drug Administration (FDA) and the European Medicines Agency (EMA) [12,13] and has proved to be effective and well tolerated in prospective and retrospective studies [14,15,16,17,18,19,20]. For patients who present or develop resistance to this form of PSMA-RLT, further treatment options are very limited. In this setting, the use of an alpha emitter, e.g., in the form of [^225^Ac]Ac-PSMA-617, may serve as a therapy-escalating option to intensify the anti-tumor effect. Alpha emitters such as ^225^Ac induce a higher rate of double-strand breaks in the DNA of target cells, which are less likely to be repaired by the cell’s DNA-repair system than the single-strand breaks, which are commonly induced by ^177^Lu [21]. Besides the increased anti-tumor effect, increased side effects, especially therapy limiting xerostomia, are observed when applying [^225^Ac]Ac-PSMA-617 as a monotherapy [22,23].

One approach to balance adverse events and the anti-tumor effect is the combined application of [^177^Lu]Lu-PSMA-617 and [^225^Ac]Ac-PSMA-617 with adjusted doses, known as the ‘PSMA tandem RLT’, which has been shown to be effective, especially in this challenging patient cohort [24,25,26,27,28]. This form of therapy has recently been introduced and has so far only been studied clinically in small cohorts of patients. There is limited research on this topic and, to our knowledge, only two studies have investigated potential biomarkers associated with this treatment [25,28]. However, the utilization of biomarkers is crucial for assessing the expected therapeutic benefit for the individual patient and for objective monitoring during treatment. While for [^177^Lu]Lu-PSMA-617 monotherapy, ^18^F-Flurodeoxyglucose ([^18^F]FDG) positron emission tomography (PET)/computed tomography (CT) has been shown to be an additional valuable tool for predicting response to treatment [29,30], such reports are still missing in the context of [^225^Ac]Ac-PSMA-617/[^177^Lu]Lu-PSMA-617 tandem therapy. In this study, we evaluated the utility of [^18^F]FDG PET/CT derived molecular imaging biomarkers for predicting response and outcome to PSMA tandem RLT in patients with insufficient response on [^177^Lu]Lu-PSMA-617 monotherapy.

## 2. Materials and Methods

### 2.1. Patient Population and Study Design

**Figure 1:**
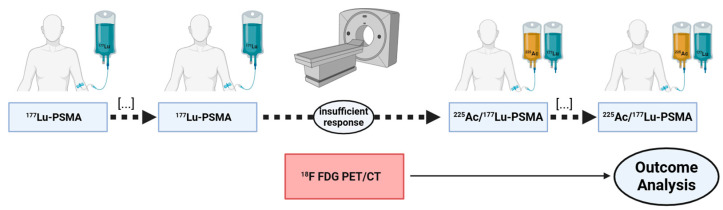
Schematic summary of study design.

This study retrospectively analyzed a cohort of *n* = 33 mCRPC patients who received [^177^Lu]Lu-PSMA-617 RLT, augmented by at least one cycle of [^225^Ac]Ac-PSMA-617. Prior to this ^225^Ac augmented PSMA-RLT, patients had received up to eight cycles of [^177^Lu]Lu-PSMA-617 monotherapy (range: 1–8 cycles). All of the patients enrolled showed insufficient response on [^177^Lu]Lu-PSMA-617 monotherapy, defined as any increase in prostate-specific antigen (PSA) or a decrease < 50%. In an attempt to analyze molecular imaging biomarkers in [^18^F]FDG PET/CT, the inclusion criteria required that all included patients had received [^18^F]FDG PET/CT scans within one month prior to the first cycle of [^225^Ac]Ac-PSMA-617/[^177^Lu]Lu-PSMA-617 tandem RLT. A visual representation of the study design is shown in Figure 1. All patients received intensive pretreatment prior to any RLT and intense PSMA expression was verified by [^68^Ga]Ga-PSMA-11 PET/CT. Details of patient pretreatment and characteristics are given in Table 1. Informed consent was obtained from all patients included in this study in accordance with the Declaration of Helsinki. PSMA-RLT was applied on a compassionate use basis, following the regulations of the German Pharmaceutical Act §13 (2b). The analysis was approved by the local institutional review board (ethics committee approval number 140/17).

### 2.2. Details of PSMA-RLT

The mean time interval between discontinuation of [^177^Lu]Lu-PSMA-617 monotherapy and initiation of [^225^Ac]Ac-PSMA-617 augmented [^177^Lu]Lu-PSMA-617 RLT was 2 ± 2 months. During the tandem PSMA-RLT, patients received a mean of 2 ± 1 cycles of [^177^Lu]Lu-PSMA-617 and a mean of 2 ± 1 augmentations with [^225^Ac]Ac-PSMA-617. The mean administered activity per cycle was 5.4 ± 1.7 GBq for [^177^Lu]Lu-PSMA-617 and 3.8 ± 1.7 MBq for [^225^Ac]Ac-PSMA-617. The mean cumulative activity was 9.4 ± 6.4 GBq for [^177^Lu]Lu-PSMA-617 and 7.3 ± 6.7 MBq for [^225^Ac]Ac-PSMA-617. The administered [^177^Lu]Lu-PSMA-617 and [^225^Ac]Ac-PSMA-617 were synthesized according to the published procedures of Kratochwil et al. [31,32]. The PSMA-617 was provided by ABX Advanced Biochemical Compounds GmbH (Radeberg, Germany). ^177^Lu was provided by Eczacıbaşı-Monrol Nuclear Products Co. (Istanbul, Turkey), while ^225^Ac was obtained from Van Overeem Nuclear b.v. (Breda, The Netherlands). For 6 GBq of ^177^Lu, 150 µg of PSMA-617 was used for labeling, while for 10 MBq of ^225^Ac, 300 µg of PSMA-617 was applied. The injected activities were adapted for each individual patient, depending on the characteristics of body surface area, the assessed tumor burden (in [^18^F]FDG PET/CT and [^68^Ga]Ga-PSMA-11 PET/CT), the presence of metastases located in the bone marrow, the grade of renal impairment, and the course of disease. In agreement with the German Radiation Protection Act, all patients were treated under the conditions of an inpatient stay. Intravenous hydration (500 mL 0.9% NaCl solution) was given to all patients, starting 30 min prior to injection of the radiopharmaceutical. In addition, salivary gland cooling was administered to the patients.

### 2.3. [^18^F]FDG PET/CT Image Acquisition

Patients underwent [^18^F]FDG PET/CT scans within one month prior to the initiation of tandem RLT. The mean cumulative administered activity was 264 ± 42 MBq. Following tracer injection, patients received an infusion of 500 mL of 0.9% NaCl. [^18^F]FDG was provided by ZAG (Karlsruhe, Germany). In accordance with the current imaging guidelines [33], the interval between tracer injection and imaging was 60 min. The PET/CT scans were performed in 3D-ToF mode, utilizing a Biograph 40 mCT PET/CT scanner (Siemens Medical Solutions, Knoxville, TN, USA), applying an extended field of view of 21.4 cm (TrueV). The acquisition of PET images was performed between vertex and mid-femur with a 2 min/bed position. The slice thickness was 3.00 mm and a pixel matrix of 200 × 200 was applied. Attenuation-corrected PET reconstruction was performed using a three-dimensional OSEM algorithm with Gaussian filtering, 3 iterations, 21 subsets, and a reconstructed slice thickness of 5.0 mm. Additionally, scatter correction, decay correction, and random correction were applied. For attenuation correction and anatomic localization, low-dose CT was performed, using an X-ray tube voltage of 120 keV and a modulation of the tube current using CARE Dose4D with a reference tube current of 30 mAs. All CT scans were reconstructed within a 200 × 200 matrix, employing a slice thickness of 5.0 mm and an increment of 2.0–4.0 mm.

### 2.4. Evaluation of Predictive Biomarkers

**Figure 2:**
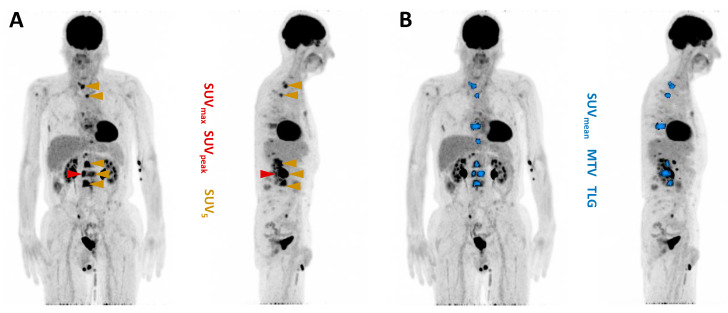
Maximum intensity projection of [^18^F]FDG PET/CT with illustration of PET-derived parameters. (**A**) SUV_max_ (red), SUV_peak_ (red), SUV_5_ (gold). (**B**) SUV_mean_ (blue), MTV (blue), TLG (blue).

[^18^F]FDG PET/CT images were analyzed using the Syngo.Via software (Enterprise, software version number VB 60, Siemens, Erlangen, Germany) and the following parameters were calculated: (i) SUV_max_, (ii) SUV_peak_, (iii) SUV_mean_ of the five most intense lesions (SUV_5_), (iv) SUV_mean_ of all lesions, (v) the whole-body metabolic tumor volume (MTV), and (vi) the total lesion glycolysis (TLG) [33,34]. MTV and TLG were evaluated by applying a semi-automatic tumor segmentation algorithm using 41% SUV_max_ as the threshold [35]. Figure 2 exemplifies the derived parameters. All parameters were tested for their association with patients’ biochemical response and overall survival (OS). Following the recommendations of the ‘prostate cancer clinical trials working group 3′ (PCWG 3) [36], progressive disease (PD) was defined as a serum PSA increase of ≥25%, while a partial remission (PR) was defined as a serum PSA decrease of ≥50%. Patients with an increase in serum PSA level of <25% or a decrease of <50% were considered to have stable disease (SD). Patients with PR were categorized as responders and patients with SD or PD as non-responders.

### 2.5. Statistical Analysis

PRISM 10 software (GraphPad Software, San Diego, CA, USA) was used for statistical analysis. The threshold for statistical significance was set at *p* < 0.05. OS was defined as the time interval between the start of [^225^Ac]Ac-PSMA-617-augmented [^177^Lu]Lu-PSMA-617 RLT and either the occurrence of death from any cause or the last contact with the patient. The Mann–Whitney U test was used to determine the association between each biomarker and response. The Kaplan–Meier method was used to test for association with OS, with groups stratified by the respective median value.

## 3. Results

**Figure 3:**
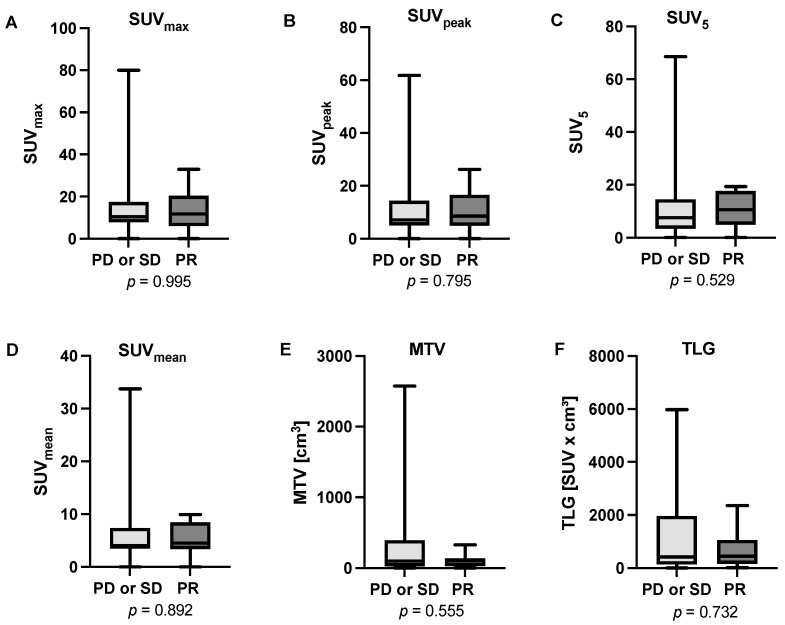
Boxplots presenting the analyzed parameters derived from [^18^F]FDG PET/CT prior to the initiation of tandem PSMA-RLT (**A**) SUV_max_, (**B**) SUV_peak_, (**C**) SUV_5_, (**D**) SUV_mean_, (**E**) MTV, and (**F**) TLG. None of the parameters showed a significant difference between responders (patients showing partial remission, PR) and non-responders (patients showing stable disease, SD or progressive disease, PD).

The analysis of biochemical response to [^225^Ac]Ac-PSMA-617-augmented [^177^Lu]Lu-PSMA-617 RLT revealed a median pre-therapeutic PSA value of 98 ng/mL (range: 8–2307 ng/mL) and a median post-therapeutic value of 188 ng/mL (range: 2–1865 ng/mL). In total, eight patients (24.2%) were categorized as PR and were consequently assessed as responders to the therapy. In total, 13 patients (39.4%) presented SD and 12 patients (36.4%) PD, summing up to 25 cases (75.8%) of non-responders. Based on this categorization we analyzed six different molecular imaging baseline parameters, derived from [^18^F]FDG PET/CT performed prior to initiation of tandem PSMA-RLT, revealing that none of them showed significant differences between responders and non-responders (SUV_max_: *p* = 0.995; SUV_peak_: *p* = 0.795; SUV_5_: *p* = 0.529; SUV_mean_: *p* = 0.892; MTV: *p* = 0.555; TLG: *p* = 0.732). Figure 3 shows boxplots depicting the distribution of data regarding all analyzed parameters, split by responders (PR) and non-responders (SD or PD), respectively.

**Figure 4:**
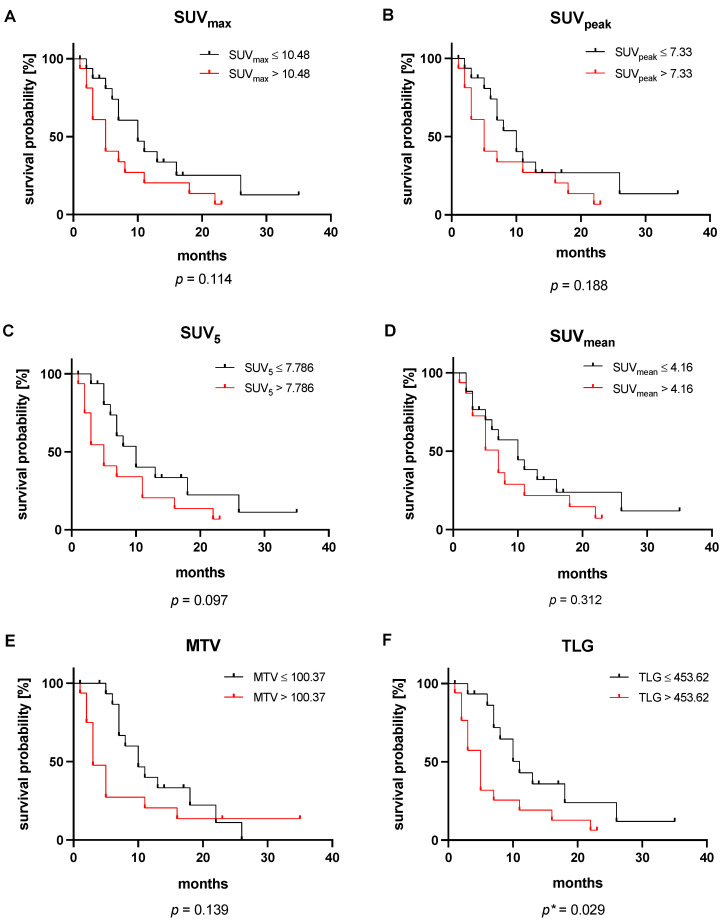
Kaplan–Meier analyses of the [^18^F]FDG PET/CT-derived parameters (**A**) SUV_max_, (**B**) SUV_peak_, (**C**) SUV_5_, (**D**) SUV_mean_, (**E**) MTV, and (**F**) TLG. All values are stratified by their respective median. * This *p*-value reached statistical significance.

The median OS of the entire cohort was 7 months (95% CI: 4–11 months). The subsequent Kaplan–Meier analysis showed no significant association with OS for five out of six analyzed parameters. Stratified by the respective median, neither SUV_max_ (*p* = 0.114), SUV_peak_ (*p* = 0.188), SUV_5_ (*p* = 0.097), SUV_mean_ (*p* = 0.312), nor MTV (*p* = 0.139) reached a level of significance. In contrast, TLG achieved a level of significance in Kaplan–Meier analysis (*p* = 0.029). Patients with a TLG ≤ 453.62 SUV x mL (median value) experienced a median of 11 months (95% CI: 7–15 months), a significantly longer OS than patients with TLG > 453.62 SUV x mL surviving a median of 5 months (95% CI: 3–7 months). The corresponding Kaplan–Meier graphs are presented in Figure 4.

**Figure 5:**
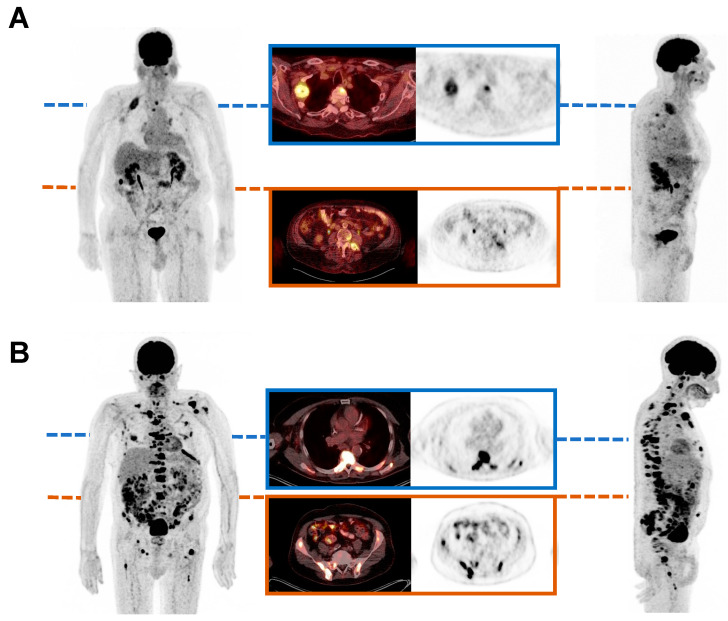
Maximum intensity projections of [^18^F]FDG PET/CT, showing two exemplary mCRPC patients before initiation of PSMA tandem RLT. The first patient showed a relatively low baseline TLG value of 287.55 SUV x mL and experienced an overall survival (OS) of 22 months (**A**). In comparison, the second patient presented a comparably high baseline TLG value of 1039.52 SUV x mL and exhibited an OS of 2 months (**B**).

In Figure 5, two exemplary patients are shown, representing the association between baseline TLG value and OS. The patient shown in Figure 5A presents a relatively low baseline TLG value, corresponding with a high OS. In contrast, Figure 5B presents a patient with a comparably high baseline TLG value and a notably shorter OS.

## 4. Discussion

[^225^Ac]Ac-PSMA-617-augmented [^177^Lu]Lu-PSMA-617 RLT is a promising therapy option in the treatment of mCRPC [24,25,26,27,28]. This bimodular form of PSMA-RLT, known as the tandem approach, was shown to be effective for patients, who experience progression of disease or insufficient response under [^177^Lu]Lu-PSMA-617 monotherapy [24,25]. This study identified baseline total lesion glycolysis (TLG) as a whole-body quantitative imaging marker predicting OS in this setting.

The here-reported pilot study including *n* = 33 mCRPC patients is, to the best of our knowledge, the first study investigating molecular imaging biomarkers derived from [^18^F]FDG PET/CT in patients receiving PSMA tandem RLT after insufficient response to conventional PSMA-RLT. As reported in prior studies on tandem RLT, the data confirm the effectiveness of this approach in patients with insufficient response under [^177^Lu]Lu-PSMA-617 monotherapy (PR in 24.2% of patients). However, none of the tested parameters (SUV_max_, SUV_peak_, SUV_5_, SUV_mean_, MTV, and TLG) were able to predict the response. This observation was somewhat expected and is in line with our clinical experience. In contrast to the prediction of response, one of the analyzed parameters, TLG, was able to predict overall survival (OS) in our cohort (*p* = 0.029). The whole-body parameter TLG, including total tumor volume and uptake, i.e., glucometabolic activity, represents a molecular imaging parameter that indicates total tumor burden and aggressiveness simultaneously, which may account for its superiority in predicting OS over other variables tested.

The suitability of TLG as a predictive imaging biomarker in prostate cancer is also broadly supported by previously published studies. Our results are in line with the work published by Ferdinandus et al., stating that [^18^F]FDG-positive tumor volume is predictive for OS in mCRPC patients undergoing conventional PSMA-RLT with [^177^Lu]Lu-PSMA-617 [37]. The finding that TLG predicts OS in mCRPC is also supported by several other studies involving different mCRPC treatments: Wibmer et al. demonstrated, in univariate and multivariable analysis, that whole-body TLG was significantly associated with OS in the context of first-line abiraterone and enzalutamide treatment [38]. Similarly, Güzel et al. have shown that TLG (labeled TTL-G in this publication) is predictive of OS for mCRPC patients undergoing taxane-based therapy [39]. Bauckneht et al. also highlighted the role of [^18^F]FDG PET as a tool for patient selection and response assessment in mCRPC patients undergoing bone-seeking ^223^Ra irradiation [40] and in another study, demonstrated that a lower TLG value was associated with higher response rates to androgen-receptor targeted agents (enzalutamide and abiraterone) [34]. It should also be noted that TLG has been identified as a predictive biomarker not only in mCRPC but also in other tumor entities, mainly lymphoma and various solid tumors [41,42,43]. Further studies are needed to determine the relevance of these FDG-derived parameters in mCPRC. Considering the results presented here, we suggest that [^18^F]FDG PET/CT should be established in the management of PSMA-RLT in clinical practice, not only to detect mismatch metastases (intense glucometabolic lesions with no or only faint PSMA expression) [44,45] but also to improve outcome prediction by assessing the whole-body molecular imaging parameter TLG. The identification and implementation of prognostic biomarkers may optimize treatment and provide a route to more individualized management in mCRPC. The implementation of TLG determination may be hampered by the time-consuming segmentation process, which can take up to half an hour per patient. However, in the future, artificial intelligence-based algorithms will certainly be able to perform TLG segmentation more efficiently, allowing for practical implementation in clinical routines.

The study is subject to certain limitations, including its retrospective monocentric character, the limited sample size, and patient selection, which may confine the interpretation and generalization of the results. Subsequent studies are necessary to confirm and extend our findings, ideally in a prospective manner and additionally across other patient cohorts in different clinical settings. While this study focused exclusively on the described tandem-RLT approach, an evaluation of FDG-derived parameters in the context of ^225^Ac-PSMA monotherapy, which was also shown to be a viable therapy option [46], is still pending. It should also be mentioned that in this investigation, we concentrated only on [^18^F]FDG PET/CT-derived parameters. Combined FDG/PSMA parameters or the impact of the presence of, for example, small-volume mismatch lesions (intense glucometabolic lesions with no or only faint PSMA expression) are certainly worth evaluating in future studies.

## 5. Conclusions

The [^18^F]FDG PET/CT-derived molecular imaging parameter of total lesion glycolysis is significantly associated with the overall survival of mCRPC patients undergoing [^225^Ac]Ac-PSMA-617-augmented [^177^Lu]Lu-PSMA-617 radioligand therapy after insufficient response to [^177^Lu]Lu-PSMA-617 monotherapy. Implementing [^18^F]FDG PET/CT in the management of PSMA-RLT in clinical practice may provide a prognostic tool and a route to more individualized management in mCRPC.

## Figures and Tables

**Table 1:** Patient characteristics.

Age	Median [years] (range)	71 (58–85)
ECOG performance score category	% (*n*)	
≤1		76 (25)
2		21 (7)
3		3 (1)
PSA	Median [ng/mL] (range)	98 (8–2307)
ALP	Median [U/L] (range)	127 (48–421)
Hemoglobin	Median [g/dL] (range)	11 (7–14)
Prior therapies	% (*n*)	
Prostatectomy		39 (13)
Radiation		52 (17)
Abiraterone		79 (26)
Enzalutamide		82 (27)
Abiraterone or Enzalutamide		100 (33)
Abiraterone and Enzalutamide		61 (20)
Docetaxel		91 (30)
Cabazitaxel		36 (12)
Docetaxel + Cabazitaxel		36 (12)
^223^Ra		15 (5)
Prior ^177^Lu-PSMA-617 RLT	% (*n*)	100 (33)
cycles	Median (range)	4 (1–8)
cumulative activity	Median [GBq] (range)	26.4 (7.4–60.4)
Site of metastases	% (*n*)	
Bone		91 (31)
Lymph node		56 (19)
Liver		26 (9)
Lung		9 (3)

Alkaline phosphatase (ALP); Eastern Cooperative Oncology Group (ECOG); prostate-specific antigen (PSA); radioligand therapy (RLT).

## Data Availability

The datasets used and analyzed during the present study are available from the corresponding author upon reasonable request.

## Abbreviations

ADT	Androgen deprivation therapy
ALP	Alkaline phosphatase
CI	Confidence interval
CT	Computed tomography
ECOG	Eastern Cooperative Oncology Group
Hb	Hemoglobin
mCRPC	Metastatic castration-resistant prostate cancer
MTV	Metabolic tumor volume
OS	Overall survival
PET	Positron emission tomography
PSA	Prostate-specific antigen
PSMA	Prostate-specific membrane antigen
PFS	Progression-free survival
RLT	Radioligand therapy
SUV	Standardized uptake value
SUV_max_	Maximum standardized uptake value
SUV_peak_	Peak standardized uptake value
SUV_mean_	Mean standardized uptake value
TLG	Total lesion glycolysis
